# 4-Meth­oxy­benzamidinium chloride monohydrate

**DOI:** 10.1107/S1600536812041219

**Published:** 2012-10-06

**Authors:** Simona Irrera, Gustavo Portalone

**Affiliations:** a Chemistry Department, "Sapienza" University of Rome, P.le A. Moro, 5, I-00185 Rome, Italy

## Abstract

In the cation of the title compound, C_8_H_11_N_2_O^+^·Cl^−^·H_2_O, the C—N bonds of the amidinium group are identical within experiemental error [1.305 (2) and 1.304 (2) Å], and its plane forms a dihedral angle of 25.83 (8)° with the phenyl ring. The ionic components are associated in the crystal into polymeric hydrogen-bonded supra­molecular tapes stabilized by N—H^+^⋯Cl^−^ and N—H^+^⋯Ow inter­molecular hydrogen bonds, and by Ow—H⋯Cl^−^ inter­actions.

## Related literature

For the biological and pharmacological relevance of benzamid­ine, see: Marquart *et al.* (1983 ▶); Sprang *et al.* (1987 ▶); Bode *et al.* (1990 ▶); Powers & Harper (1999 ▶); Grzesiak *et al.* (2000 ▶). For structural analysis of proton-transfer adducts containing mol­ecules of biological inter­est, see: Portalone (2011*a* ▶); Portalone & Irrera (2011 ▶). For the supra­molecular association in proton-transfer adducts containing benzamidinium cations, see; Portalone (2010 ▶, 2011*b* ▶, 2012 ▶). For hydrogen-bond motifs, see: Bernstein *et al.* (1995 ▶).
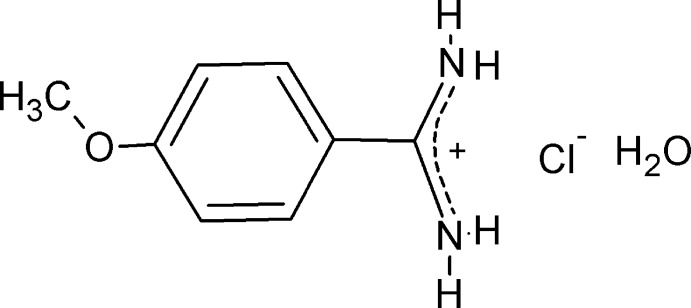


## Experimental

### 

#### Crystal data

C_8_H_11_N_2_O^+^·Cl^−^·H_2_O
*M* *_r_* = 204.65
Monoclinic, 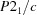

*a* = 11.3029 (7) Å
*b* = 9.3142 (5) Å
*c* = 9.9983 (6) Å
β = 99.820 (6)°
*V* = 1037.17 (11) Å^3^
*Z* = 4
Mo *K*α radiation
μ = 0.34 mm^−1^
*T* = 298 K
0.30 × 0.27 × 0.25 mm

#### Data collection

Oxford Diffraction Xcalibur S CCD diffractometer
Absorption correction: multi-scan (*CrysAlis RED*; Oxford Diffraction, 2006 ▶) *T*_min_ = 0.905, *T*_max_ = 0.968
7158 measured reflections
2989 independent reflections
1947 reflections with *I* > 2σ(*I*)
*R*_int_ = 0.018

#### Refinement

*R*[*F* ^2^ > 2σ(*F* ^2^)] = 0.043
*wR*(*F* ^2^) = 0.113
*S* = 0.97
2989 reflections
144 parameters
H atoms treated by a mixture of independent and constrained refinement
Δρ_max_ = 0.22 e Å^−3^
Δρ_min_ = −0.15 e Å^−3^

### 

Data collection: *CrysAlis CCD* (Oxford Diffraction, 2006 ▶); cell refinement: *CrysAlis CCD*; data reduction: *CrysAlis RED* (Oxford Diffraction, 2006 ▶); program(s) used to solve structure: *SIR97* (Altomare *et al.*, 1999 ▶); program(s) used to refine structure: *SHELXL97* (Sheldrick, 2008 ▶); molecular graphics: *ORTEP-3 for Windows* (Farrugia, 1997 ▶); software used to prepare material for publication: *WinGX* (Farrugia, 1999 ▶).

## Supplementary Material

Crystal structure: contains datablock(s) global, I. DOI: 10.1107/S1600536812041219/qm2085sup1.cif

Structure factors: contains datablock(s) I. DOI: 10.1107/S1600536812041219/qm2085Isup2.hkl

Additional supplementary materials:  crystallographic information; 3D view; checkCIF report

## Figures and Tables

**Table 1:** Hydrogen-bond geometry (Å, °)

*D*—H⋯*A*	*D*—H	H⋯*A*	*D*⋯*A*	*D*—H⋯*A*
N1—H1*A*⋯Cl1	0.81 (2)	2.85 (2)	3.5523 (18)	145 (2)
N1—H1*B*⋯O2*W*	0.75 (2)	2.06 (2)	2.805 (2)	168 (2)
N2—H2*A*⋯Cl1	0.96 (2)	2.25 (2)	3.1850 (16)	164.4 (16)
N2—H2*B*⋯Cl1^i^	0.82 (2)	2.43 (2)	3.201 (2)	157.5 (18)
O2*W*—H*WA*⋯Cl1^ii^	0.82 (3)	2.36 (3)	3.1731 (18)	170 (3)
O2*W*—H*WB*⋯Cl1^iii^	0.87 (2)	2.29 (2)	3.1603 (17)	174.8 (19)

Symmetry codes: (i) 
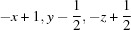
; (ii) 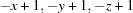; (iii) 
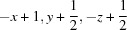
.

**Fig. 1.**
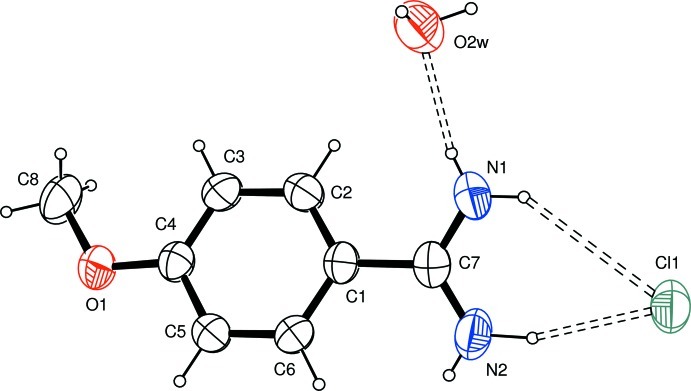
The asymmetric unit of (I), showing the atom-labelling scheme. Displacements ellipsoids are at the 50% probability level. H atoms are shown as small spheres of arbitrary radii.

**Fig. 2.**
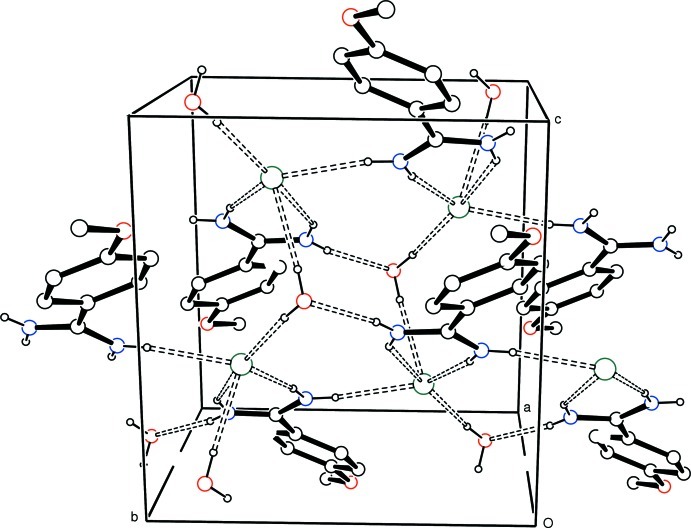
Crystal packing diagram for (I), viewed approximately down a. Displacements ellipsoids are at the 50% probability level. H atoms are shown as small spheres of arbitrary radii. For the sake of clarity, H atoms not involved in hydrogen bonding have been omitted. Hydrogen bonding is indicated by dashed lines.

## supplementary crystallographic information

### Comment

This Laboratory is currently engaged in systematic structural analysis of proton-transfer adducts containing molecules of biological interest (Portalone, 2011*a*; Portalone & Irrera, 2011). In this context benzamidine derivatives, which have shown strong biological and pharmacological activity (Powers & Harper, 1999; Grzesiak *et al.*, 2000), are being used in our group as bricks for supramolecular construction (Portalone, 2010, 2011*b*, 2012). Indeed, these molecules are strong Lewis bases and their cations can be easily anchored onto numerous inorganic and organic anions and polyanions, largely because of the presence of four potential donor sites for hydrogen-bonding. Benzamidinium ions have also been included in a number of protein structures (Marquart *et al.*, 1983; Sprang *et al.*, 1987; Bode *et al.*, 1990).

The asymmetric unit of (I) comprises one non-planar 4-methoxybenzamidinium cation, one chloride anion and one water molecule of crystallization (Fig. 1).

In the cation the amidinium group forms a dihedral angle of 25.83 (8)° with the mean plane of the phenyl ring, which agrees with the values observed in protonated benzamidinium ions (23.2 - 30.4°, Portalone, 2010, 2012). The lack of planarity in all these systems is obviously caused by steric hindrances between the H atoms of the aromatic ring and the amidine moiety. This conformation is rather common in benzamidinium-containing small-molecule crystal structures, with the exception of benzamidinium diliturate, where the benzamidinium cation is planar (Portalone, 2010). The pattern of bond lengths and bond angles of the 4-methoxybenzamidinium cation agrees with that reported in previous structural investigations (Portalone, 2010, 2012). In particular the amidinium group, true to one's expectations, features similar C—N bonds [1.305 (2) and 1.304 (2) Å], evidencing the delocalization of the π electrons and their double-bond character.

Analysis of the crystal packing of (I), (Fig. 2), shows that the ions are associated in the crystal by six distinct N—H^+^···Cl^-^, N—H^+^···Ow and Ow—H···Cl^-^ intermolecular hydrogen bonds (Table 1). Each amidinium unit is bound to two chloride anions by three weak hydrogen bonds (N^+^···Cl^-^ = 3.185 (2) - 3.552 (2) Å) and to one water molecule by one N—H^+^···Ow interaction, just forming two *R*^3^_5_(10) and one *R*^1^_2_(6) supramolecular synthons (Bernstein *et al.*, 1995). Both of these *R*^3^_5_(10) and *R*^1^_2_(6) motifs lead to the formation of one dimensional polymeric hydrogen-bonded supramolecular tapes approximately along the crystallographic *b* axis. The water molecule, which plays a dual role as both donor and acceptor in hydrogen bonding interactions, acts as a bridge between tapes through the remaining two Ow—H···Cl^-^ intermolecular interactions.

### Experimental

4-methoxybenzamidine (1 mmol, Fluka at 96% purity) was dissolved without further purification in 6 ml of hot water and heated under reflux for 3 h. While stirring, HCl (6 mol *L*^-1^) was added dropwise until the pH reached 2. After cooling the solution to ambient temperature, colourless crystals suitable for single-crystal X-ray diffraction were grown by slow evaporation of the solvent over two weeks.

### Refinement

All H atoms were identified in difference Fourier maps, but for refinement all C-bound H atoms were placed in calculated positions, with C—H = 0.93 Å (phenyl) and 0.96 Å (methyl), and refined as riding on their carrier atoms. The *U*_iso_ values were kept equal to 1.2*U*_eq_(C, phenyl). and to 1.5*U*_eq_(C, methyl). Positional and thermal parameters of H atoms of the amidinium group and of water molecule were freely refined, giving N—H distances in the range 0.75 (2) - 0.96 (2) Å and O—H distances in the range 0.82 (3) - 0.87 (2) Å

### Crystal data

**Table:**

C_8_H_11_N_2_O^+^·Cl^−^·H_2_O	*F*(000) = 432
*M**_r_* = 204.65	*D*_x_ = 1.311 Mg m^−^^3^
Monoclinic, *P*2_1_/*c*	Mo *K*α radiation, λ = 0.71069 Å
Hall symbol: -P 2ybc	Cell parameters from 3182 reflections
*a* = 11.3029 (7) Å	θ = 2.8–32.2°
*b* = 9.3142 (5) Å	µ = 0.34 mm^−^^1^
*c* = 9.9983 (6) Å	*T* = 298 K
β = 99.820 (6)°	Tablets, colourless
*V* = 1037.17 (11) Å^3^	0.30 × 0.27 × 0.25 mm
*Z* = 4	

### Data collection

**Table:**

Oxford Diffraction Xcalibur S CCD diffractometer	2989 independent reflections
Radiation source: Enhance (Mo) X-ray Source	1947 reflections with *I* > 2σ(*I*)
Graphite monochromator	*R*_int_ = 0.018
Detector resolution: 16.0696 pixels mm^-1^	θ_max_ = 30.0°, θ_min_ = 2.9°
ω and φ scans	*h* = −15→13
Absorption correction: multi-scan (*CrysAlis RED*; Oxford Diffraction, 2006)	*k* = −13→13
*T*_min_ = 0.905, *T*_max_ = 0.968	*l* = −14→14
7158 measured reflections	

### Refinement

**Table:**

Refinement on *F*^2^	Primary atom site location: structure-invariant direct methods
Least-squares matrix: full	Secondary atom site location: difference Fourier map
*R*[*F*^2^ > 2σ(*F*^2^)] = 0.043	Hydrogen site location: inferred from neighbouring sites
*wR*(*F*^2^) = 0.113	H atoms treated by a mixture of independent and constrained refinement
*S* = 0.97	*w* = 1/[σ^2^(*F*_o_^2^) + (0.0667*P*)^2^] where *P* = (*F*_o_^2^ + 2*F*_c_^2^)/3
2989 reflections	(Δ/σ)_max_ < 0.001
144 parameters	Δρ_max_ = 0.22 e Å^−^^3^
0 restraints	Δρ_min_ = −0.15 e Å^−^^3^

### Special details

**Table:**

Geometry. All s.u.'s (except the s.u. in the dihedral angle between two l.s. planes) are estimated using the full covariance matrix. The cell s.u.'s are taken into account individually in the estimation of s.u.'s in distances, angles and torsion angles; correlations between s.u.'s in cell parameters are only used when they are defined by crystal symmetry. An approximate (isotropic) treatment of cell s.u.'s is used for estimating s.u.'s involving l.s. planes.
Refinement. Refinement of *F*^2^ against ALL reflections. The weighted *R*-factor *wR* and goodness of fit *S* are based on *F*^2^, conventional *R*-factors *R* are based on *F*, with *F* set to zero for negative *F*^2^. The threshold expression of *F*^2^ > 2σ(*F*^2^) is used only for calculating *R*-factors(gt) *etc*. and is not relevant to the choice of reflections for refinement. *R*-factors based on *F*^2^ are statistically about twice as large as those based on *F*, and *R*- factors based on ALL data will be even larger.

### Fractional atomic coordinates and isotropic or equivalent isotropic displacement parameters (Å^2^)

**Table:**

	*x*	*y*	*z*	*U*_iso_*/*U*_eq_	
Cl1	0.59874 (3)	0.29686 (5)	0.19860 (4)	0.06143 (17)	
O1	0.02187 (9)	0.02872 (12)	0.72746 (11)	0.0585 (3)	
N1	0.37553 (14)	0.36637 (18)	0.40096 (17)	0.0577 (4)	
H1A	0.4215 (18)	0.391 (3)	0.351 (2)	0.087 (7)*	
H1B	0.3457 (16)	0.424 (2)	0.4357 (19)	0.070 (7)*	
N2	0.40806 (13)	0.1372 (2)	0.34687 (16)	0.0609 (4)	
H2A	0.4716 (18)	0.167 (2)	0.300 (2)	0.082 (6)*	
H2B	0.3898 (16)	0.052 (3)	0.3475 (19)	0.077 (7)*	
C1	0.26509 (11)	0.18152 (15)	0.49586 (13)	0.0400 (3)	
C2	0.17291 (12)	0.26955 (17)	0.52024 (16)	0.0499 (4)	
H2	0.1669	0.3620	0.4847	0.060*	
C3	0.08913 (12)	0.22277 (17)	0.59666 (16)	0.0497 (4)	
H3	0.0273	0.2832	0.6120	0.060*	
C4	0.09816 (11)	0.08562 (16)	0.64994 (14)	0.0425 (3)	
C5	0.19078 (12)	−0.00346 (17)	0.62752 (15)	0.0489 (4)	
H5	0.1974	−0.0953	0.6645	0.059*	
C6	0.27322 (12)	0.04365 (16)	0.55054 (14)	0.0460 (3)	
H6	0.3348	−0.0171	0.5350	0.055*	
C7	0.35238 (11)	0.22994 (17)	0.41160 (14)	0.0460 (4)	
C8	−0.07725 (14)	0.1140 (2)	0.75071 (19)	0.0684 (5)	
H8A	−0.0482 (3)	0.2006 (11)	0.7969 (13)	0.103*	
H8B	−0.1241 (9)	0.0612 (8)	0.8055 (12)	0.103*	
H8C	−0.1263 (8)	0.1375 (12)	0.6654 (9)	0.103*	
O2W	0.27853 (14)	0.61140 (16)	0.50438 (18)	0.0783 (4)	
HWA	0.319 (3)	0.631 (4)	0.579 (3)	0.155 (14)*	
HWB	0.3124 (17)	0.667 (2)	0.452 (2)	0.077 (6)*	

### Atomic displacement parameters (Å^2^)

**Table:**

	*U*^11^	*U*^22^	*U*^33^	*U*^12^	*U*^13^	*U*^23^
Cl1	0.0604 (3)	0.0699 (3)	0.0594 (3)	−0.00364 (19)	0.02536 (19)	0.0085 (2)
O1	0.0518 (6)	0.0581 (7)	0.0737 (7)	0.0039 (5)	0.0337 (5)	0.0110 (6)
N1	0.0595 (8)	0.0550 (9)	0.0642 (9)	−0.0047 (7)	0.0267 (7)	0.0125 (8)
N2	0.0583 (8)	0.0688 (11)	0.0628 (9)	−0.0090 (8)	0.0311 (7)	−0.0027 (8)
C1	0.0358 (6)	0.0472 (8)	0.0373 (7)	−0.0039 (6)	0.0072 (5)	0.0001 (6)
C2	0.0510 (8)	0.0403 (8)	0.0604 (9)	0.0036 (6)	0.0156 (7)	0.0089 (7)
C3	0.0437 (7)	0.0463 (9)	0.0629 (9)	0.0077 (6)	0.0195 (6)	0.0020 (7)
C4	0.0384 (6)	0.0464 (8)	0.0443 (7)	−0.0017 (6)	0.0117 (5)	0.0002 (6)
C5	0.0496 (7)	0.0421 (8)	0.0584 (9)	0.0047 (7)	0.0191 (6)	0.0082 (7)
C6	0.0420 (7)	0.0449 (8)	0.0541 (8)	0.0078 (6)	0.0167 (6)	0.0032 (7)
C7	0.0405 (7)	0.0559 (10)	0.0420 (7)	−0.0040 (6)	0.0084 (6)	0.0045 (7)
C8	0.0503 (8)	0.0838 (13)	0.0787 (12)	0.0101 (8)	0.0329 (8)	0.0039 (10)
O2W	0.1035 (11)	0.0605 (8)	0.0747 (10)	−0.0175 (8)	0.0258 (9)	0.0015 (7)

### Geometric parameters (Å, º)

**Table:**

O1—C4	1.3613 (16)	C2—H2	0.9300
O1—C8	1.4243 (18)	C3—C4	1.381 (2)
N1—C7	1.305 (2)	C3—H3	0.9300
N1—H1A	0.81 (2)	C4—C5	1.3837 (19)
N1—H1B	0.75 (2)	C5—C6	1.3776 (19)
N2—C7	1.304 (2)	C5—H5	0.9300
N2—H2A	0.96 (2)	C6—H6	0.9300
N2—H2B	0.82 (2)	C8—H8A	0.9596
C1—C2	1.3798 (19)	C8—H8B	0.9596
C1—C6	1.3926 (19)	C8—H8C	0.9596
C1—C7	1.4731 (18)	O2W—HWA	0.82 (3)
C2—C3	1.385 (2)	O2W—HWB	0.87 (2)
			
C4—O1—C8	117.92 (13)	C3—C4—C5	120.02 (12)
C7—N1—H1A	119.0 (17)	C6—C5—C4	120.14 (14)
C7—N1—H1B	123.3 (15)	C6—C5—H5	119.9
H1A—N1—H1B	118 (2)	C4—C5—H5	119.9
C7—N2—H2A	120.7 (11)	C5—C6—C1	120.49 (12)
C7—N2—H2B	119.6 (14)	C5—C6—H6	119.8
H2A—N2—H2B	119.7 (18)	C1—C6—H6	119.8
C2—C1—C6	118.68 (12)	N2—C7—N1	118.95 (15)
C2—C1—C7	121.24 (13)	N2—C7—C1	120.53 (15)
C6—C1—C7	120.07 (12)	N1—C7—C1	120.52 (15)
C1—C2—C3	121.24 (14)	O1—C8—H8A	109.5
C1—C2—H2	119.4	O1—C8—H8B	109.5
C3—C2—H2	119.4	H8A—C8—H8B	109.5
C4—C3—C2	119.43 (13)	O1—C8—H8C	109.5
C4—C3—H3	120.3	H8A—C8—H8C	109.5
C2—C3—H3	120.3	H8B—C8—H8C	109.5
O1—C4—C3	124.57 (12)	HWA—O2W—HWB	100 (3)
O1—C4—C5	115.41 (13)		
			
C6—C1—C2—C3	−0.4 (2)	C3—C4—C5—C6	−0.9 (2)
C7—C1—C2—C3	178.39 (14)	C4—C5—C6—C1	0.7 (2)
C1—C2—C3—C4	0.2 (2)	C2—C1—C6—C5	−0.1 (2)
C8—O1—C4—C3	2.8 (2)	C7—C1—C6—C5	−178.88 (14)
C8—O1—C4—C5	−178.22 (14)	C2—C1—C7—N2	−153.81 (15)
C2—C3—C4—O1	179.33 (14)	C6—C1—C7—N2	25.0 (2)
C2—C3—C4—C5	0.4 (2)	C2—C1—C7—N1	26.4 (2)
O1—C4—C5—C6	−179.90 (14)	C6—C1—C7—N1	−154.78 (15)

### Hydrogen-bond geometry (Å, º)

**Table:**

*D*—H···*A*	*D*—H	H···*A*	*D*···*A*	*D*—H···*A*
N1—H1*A*···Cl1	0.81 (2)	2.85 (2)	3.5523 (18)	145 (2)
N1—H1*B*···O2*W*	0.75 (2)	2.06 (2)	2.805 (2)	168 (2)
N2—H2*A*···Cl1	0.96 (2)	2.25 (2)	3.1850 (16)	164.4 (16)
N2—H2*B*···Cl1^i^	0.82 (2)	2.43 (2)	3.201 (2)	157.5 (18)
O2*W*—H*WA*···Cl1^ii^	0.82 (3)	2.36 (3)	3.1731 (18)	170 (3)
O2*W*—H*WB*···Cl1^iii^	0.87 (2)	2.29 (2)	3.1603 (17)	174.8 (19)

Symmetry codes: (i) −*x*+1, *y*−1/2, −*z*+1/2; (ii) −*x*+1, −*y*+1, −*z*+1; (iii) −*x*+1, *y*+1/2, −*z*+1/2.
